# Stockton's New Style of Mineral Teeth

**Published:** 1851-04

**Authors:** 


*Stockton's New Style of Mineral Teeth.*—Mr. S. W. Stockton, of Philadelphia, has recently placed the members of the dental profession under renewed obligations to him for a very great improvement which he has recently made in the manufacture of porcelain teeth. He is now producing an article which, for beauty and every other desirable quality, surpasses any we have ever seen.

